# Internal Jugular Vein Thrombosis: A Bicentric Cohort Study

**DOI:** 10.3390/jcm14113626

**Published:** 2025-05-22

**Authors:** Andrea Boccatonda, Fabiana Di Vincenzo, Ilaria Olivieri, Damiano D’Ardes, Gianfranco Lessiani, Nicoletta Di Gregorio, Susanna Vicari, Claudio Ferri

**Affiliations:** 1 Internal Medicine, Bentivoglio Hospital, AUSL Bologna, 40010 Bologna, Italy; susannavicdam@gmail.com; 2 Department of Medical and Surgical Sciences, University of Bologna, 40138 Bologna, Italy; 3 Department of Life, Health & Environmental Sciences and Internal Medicine, ASL Avezzano-Sulmona-L’Aquila, San Salvatore Hospital, University of L’Aquila, 67100 L’Aquila, Italy; fabiana.divincenzo@graduate.univaq.it (F.D.V.); ilaria.olivieri1@graduate.univaq.it (I.O.); nicolettadigregorio@yahoo.it (N.D.G.); claudio.ferri@univaq.it (C.F.); 4 Department of Medicine and Aging Science, Institute of “Clinica Medica”, “G. D’Annunzio” University of Chieti-Pescara, 66100 Chieti, Italy; damiano.dardes@unich.it; 5 Villa Serena Hospital, 65030 Città Sant’Angelo, Italy; gf.lessiani@gmail.com

**Keywords:** internal jugular vein thrombosis, catheter-related thrombosis, biomarkers, mortality, pulmonary embolism

## Abstract

**Background:** Internal jugular vein thrombosis (IJVT) is a rare but serious complication in hospitalized patients, often associated with central venous access devices (CVADs). The primary objective of the study was to analyze the clinical characteristics of patients with newly diagnosed IJVT, in particular to evaluate mortality, development of pulmonary embolism and incidence of bleeding at 30 days from diagnosis. Secondly, a sub-analysis was performed between patients with device-related and non-device-related thrombosis. **Methods:** Prospective study on adult inpatients diagnosed with IJVT from January to December 2024. Data on demographics, comorbidities, device use, laboratory values at diagnosis (D-dimer, platelet count, C-reactive protein (CRP), liver/renal function), treatment, and outcomes (mortality, pulmonary embolism, bleeding) were collected. **Results:** Thirty-one patients with IJVT were included. Mean age was 71.0 ± 13.2 years; 54.8% female; 35.5% had CVADs (central venous catheter (CVC) 36.4%, midlines 36.4%, peripherally inserted central catheter (PICC) 27.2%). Device-associated IJVT patients exhibited lower D-dimer (2.1 ± 0.5 vs. 3.6 ± 0.8 µg/mL; *p* = 0.018), higher platelet counts (249.0 ± 86.7 vs. 184.3 ± 53.6 × 10^9^/L; *p* = 0.044), and elevated CRP (12.5 ± 9.2 vs. 5.1 ± 5.6 mg/L; *p* = 0.033). Overall mortality was 16.1%; pulmonary embolism occurred in 16.1% and bleeding in 6.5%. CVAD use was not independently associated with adverse outcomes. **Conclusions:** IJVT presents with distinct biomarker profiles when associated with CVADs, characterized by lower systemic fibrinolysis and heightened inflammation. Recognition of these differences may refine diagnostic thresholds and guide prophylactic strategies. Larger prospective studies are warranted.

## 1. Introduction

Venous thromboembolism (VTE) represents a significant clinical challenge, particularly in patients with cancer, where the risk of thrombosis is markedly increased [1,2]. The presence of malignancy has been shown to elevate the risk of VTE by four to seven times compared to the general population, making it one of the most common and serious complications encountered in oncological care [1,2,3]. While lower extremity deep vein thrombosis (DVT) is well-studied, upper extremity venous thrombosis (UEVT) and, in particular, internal jugular vein thrombosis (IJVT) remain relatively underexplored. The upper extremity venous system, including the internal jugular vein, poses unique diagnostic and therapeutic challenges, especially in cancer patients where central venous access devices (CVADs) are often utilized for chemotherapy and supportive care [4]. The use of peripherally inserted central catheters (PICCs) and chest ports, while essential for treatment delivery, significantly increases the risk of catheter-related thrombosis [4]. Among cancer patients, IJVT is an uncommon but serious event [5], with complications such as pulmonary embolism (PE), superior vena cava (SVC) syndrome, and post-thrombotic syndrome reported in up to 50% of cases [6,7]. Central venous catheters (CVCs) are a critical risk factor, often associated with endothelial injury and local inflammation, which predispose to thrombus formation [8]. Studies indicate that the complication rate is particularly high when the internal jugular vein is involved, with data suggesting that isolated jugular thrombosis carries a higher risk of adverse outcomes compared to other upper extremity veins [6,8]. Despite the recognized association between cancer, CVADs, and IJVT, the clinical course and biomarker profiles of patients with catheter-related thrombosis remain inadequately characterized. In addition, the impact of malignancy stage and other patient-specific factors on the risk of thrombosis and subsequent complications is not fully understood. Given the critical implications for patient management and prognosis, it is essential to further investigate the clinical characteristics and outcomes of IJVT in the context of malignancy and CVAD use. This study aimed to analyze the clinical characteristics of patients with newly diagnosed IJVT, in particular to evaluate mortality, development of PE and incidence of bleeding at 30 days from diagnosis. Secondly, we performed sub-analyses according to the presence of vascular devices, mortality, development of pulmonary embolism and active cancer. We also evaluated the therapeutic choices regarding the prescription and type of anticoagulant therapy.

## 2. Methods

### 2.1. Study Design and Population

We performed a bicentric prospective cohort study at the San Salvatore Hospital (L’Aquila, Italy) and the Bentivoglio Hospital (Italy). Patients were enrolled at the vascular diagnostic centers of the respective hospitals, where the echo-color-Doppler diagnosis of IJVT was made between 1 January and 31 December 2024. In addition, we reviewed electronic medical records of all adult inpatients diagnosed with IJVT. Inclusion criteria were patients aged >18 years with a new diagnosis of IJVT confirmed by an echo-color-Doppler examination. All cases of IJVT were enrolled, both those limited to the aforementioned vein and those extended by a distal venous thrombosis of the arm. Patients with a known diagnosis of IJV thrombosis were excluded. Subsequently, we performed sub-analysis among patients with device-related or non-device-related forms, among subjects who died, patients who developed pulmonary embolism, and with active oncological disease. This study was approved by the institutional review board (IRB) (CE-AVEC 0013121). This study was performed according to Good Clinical Practice regulations (Good Clinical Practice for Trial on Medicinal Product—CPMP/European Commission-July 1990; Decreto Ministeriale 27.4.1992—Ministero della Sanità) and the Declaration of Helsinki (2013). By signing the protocol consent form, study participants committed themselves to adhere to local legal requirements. Informed consent was obtained from all patients enrolled. The number of included patients corresponded to all eligible cases during the study period that met the predefined inclusion criteria. Moreover, due to the scarcity of prior studies specifically addressing IJVT and the absence of robust incidence data in similar patient populations, it was not feasible to perform a reliable a priori sample size calculation.

### 2.2. Data Collection

Collected variables included demographics, comorbidities (hypertension, diabetes, malignancy, sepsis, chronic liver disease), CVAD use (type), home treatment (anticoagulants, antiplatelets), and 30-days outcomes such as in-hospital mortality, PE, minor and major bleeding defined by International Society of Thrombosis and Haemostasis (ISTH) criteria [9]. (Major: fatal bleeding, and/or symptomatic bleeding in a critical area or organ, such as intracranial, intraspinal, intraocular, retroperitoneal, intra-articular or pericardial, or intramuscular with compartment syndrome, and/or bleeding causing a fall in hemoglobin levels of 1.24 mmol/L (20 g/L or greater) or more, or leading to a transfusion of 2 U or more of whole blood or red cells. Minor: all reported bleedings not classified as major).

In addition, laboratory parameters such as hepatic enzymes (AST, ALT), total bilirubin, and serum creatinine were also collected to allow a broader characterization of the patient population. These variables were included to assess potential systemic conditions (e.g., liver or kidney dysfunction) that might influence inflammation or coagulation pathways.

## 3. Statistical Analysis

Continuous variables were compared using Student’s *t*-test or Mann–Whitney U test; categorical variables by chi-square or Fisher’s exact test. Multivariable logistic regression identified independent predictors of mortality, PE, and bleeding. A *p*-value < 0.05 was considered statistically significant. Analyses were performed using SPSS v28.

## 4. Results

### 4.1. Baseline Characteristics

**Figure 1:**
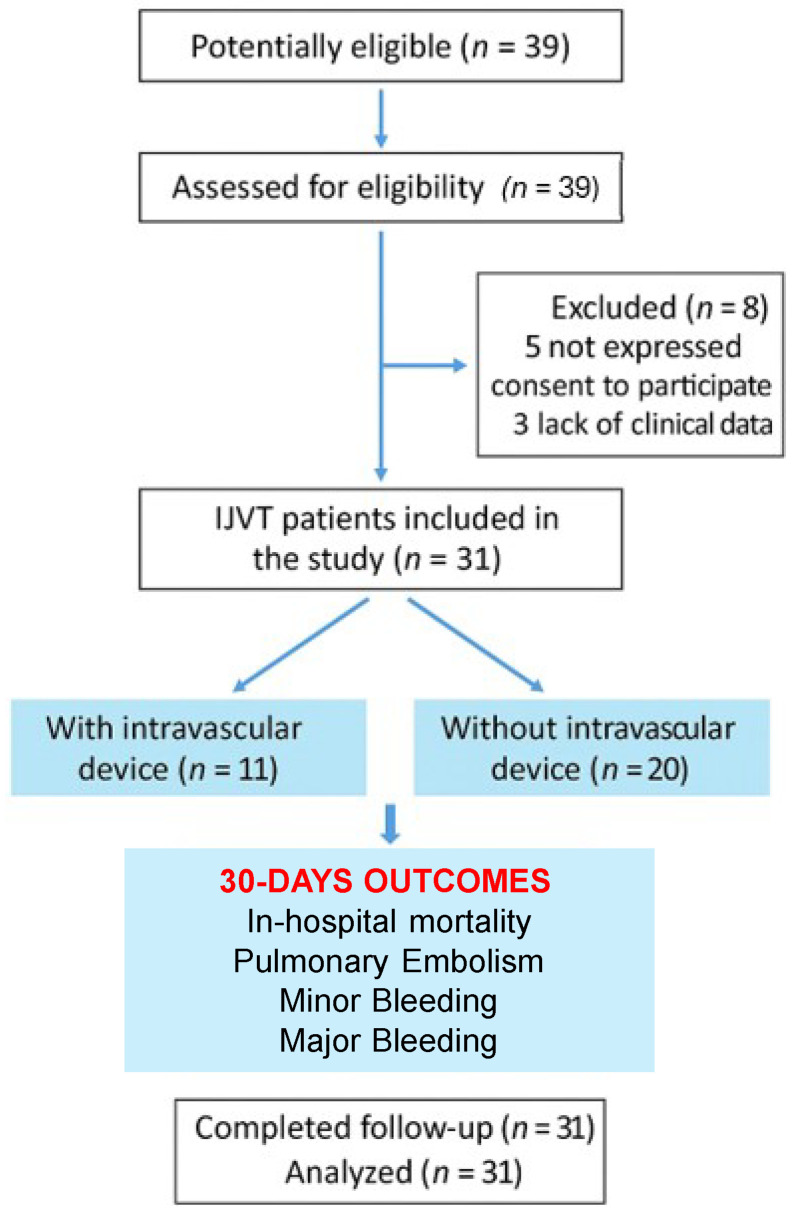
Study Flow Chart. Internal Jugular Vein Thrombosis, IJVT.

Thirty-one patients with IJVT were included (Figure 1): 17 males (54.8%) and 14 females (45.2%), mean age 71.0 ± 13.2 years. CVADs were present in 11 patients (35.5%): 4 CVCs, 4 midlines, and 3 PICCs. Active malignancy was present in 10/31 patients (32.3%), and metastatic disease in 5 cases (16.1%); sepsis in 6/31 patients (19.4%), and hypertension in 13/31 (41.9%). No patient had congenital thrombophilia; one was on oral contraceptives (Table 1). In 17 cases (54.8%), the thrombosis was associated with an upper extremity venous system thrombosis. An IJVT incidence of 8.3 cases per 1000 patients evaluated by color-Doppler examinations in the two centers was estimated.

### 4.2. Laboratory Findings

The data on the laboratory tests for the entire study cohort are shown in Table 2. In subgroup analyses, compared to patients without a CVAD, those with a CVAD had significantly lower D-dimer levels (2.1 ± 0.5 vs. 3.6 ± 0.8 µg/mL; *p* = 0.018), higher platelet counts (249.0 ± 86.7 vs. 184.3 ± 53.6 × 10^9^/L; *p* = 0.044), and elevated CRP (12.5 ± 9.2 vs. 5.1 ± 5.6 mg/L; *p* = 0.033). No significant differences were observed in other laboratory parameters. There were also no statistically significant differences between patients who died and those who survived, between those who developed PE and those who did not, or between those with active cancer and those without (Table 3, Table 4, Table 5 and Table 6).

### 4.3. Treatment

Among the 31 patients included in the analysis, there was extensive use of antithrombotic agents, albeit with varying therapeutic approaches (Table 7). In particular, low-molecular-weight heparin (LMWH) was administered to 12 patients (38.7%). As alternatives fondaparinux (FDP) and apixaban were administered to 5 patients (16.1%) and 3 patients (9.7%), respectively, whereas edoxaban was used in a single case. No prescriptions for dabigatran or rivaroxaban were observed. Vitamin K antagonist (VKA) therapy was noted in 3 patients (9.7%). Similarly, the use of acetylsalicylic acid (ASA) was reported in 3 patients (9.7%). Notably, 4 patients (12.9%) received no anticoagulant therapy. No patient was treated with unfractionated heparin (UFH).

### 4.4. Clinical Outcomes

Overall mortality was 16.1% (5/31), with no significant difference between CVAD and non-CVAD groups (18.2% vs. 14.3%; *p* = 0.78). PE occurred in 16.1% and bleeding in 6.5% (Table 8). There was only one case of cerebral hemorrhage before the diagnosis of thrombosis; two cases of bleeding post-introduction of anticoagulant therapy were detected, one minor and one major arising from a tumor mass. There were no statistically significant differences in laboratory test results between treated and untreated patients (Table 9).

## 5. Discussion

In this prospective cohort, we investigated the clinical characteristics, laboratory parameters, and clinical outcomes of patients diagnosed with IJVT. Consistent with prior literature, our findings indicate that cancer and CVADs are key contributors to thrombosis risk. Notably, one-third of our cohort had active malignancy, and CVADs were present in 35.5% of cases. Despite the recognized role of device-related endothelial injury in thrombus formation, sub-analyses did not reveal higher mortality or PE rates in the CVAD group. However, patients with a CVAD demonstrated a distinctive biomarker profile (lower D-dimer, higher platelet counts, and increased CRP), suggesting a possible localized thrombotic and inflammatory process. Moreover, the elevated CRP levels observed in patients with CVADs may reflect not only localized inflammation due to device-related endothelial injury but also a higher degree of systemic illness. Liver and kidney function markers were included to ensure that baseline systemic differences between groups did not confound the primary outcomes. Renal impairment, in particular, is known to be associated with altered inflammatory and thrombotic responses. The inclusion of these parameters supports a more comprehensive assessment of the patients’ clinical status and strengthens the validity of the observed associations between CVAD and prothrombotic laboratory findings.

Patients requiring central venous access are often more clinically fragile, with more complex medical conditions, which may independently contribute to an increased inflammatory profile. Although our study design did not include illness severity scores, this hypothesis is consistent with the clinical context and deserves further investigation.

Although it may be a known trigger [10,11], we have not recorded cases of IJVT related or secondary to heparin-induced thrombocytopenia.

Notably, there was not fully standardized thromboprophylaxis protocol universally applied across the participating institutions. However, institutional guidelines recommended risk-stratified prophylaxis based on patient comorbidities, type of admission (medical vs. surgical), and presence of central venous access devices. LMWH was the most commonly used agent, in line with national and international guidelines [12,13]. The observed variability in anticoagulant use among patients reflects individualized clinical decisions and highlights the need for more uniform thromboprophylaxis pathways in high-risk inpatients, especially those with intravascular devices.

Therapeutic regimens varied, with LMWH used most frequently, reflecting a preference for agents with a proven safety record and established efficacy in malignancy-associated thrombosis. The alternative agents FDP and apixaban were employed in five patients (16.1%) and three patients (9.7%), respectively, while edoxaban was used in only one case. No prescriptions for dabigatran or rivaroxaban were observed, highlighting a selective preference for certain direct oral anticoagulants (DOACs), likely dictated by specific clinical factors (e.g., renal function, interaction with other therapies, or tumor type). Among the four patients who were prescribed a DOAC, two were already taking it (one patient on apixaban for atrial fibrillation and one patient on apixaban for pulmonary embolism). VKA therapy was noted in three patients (9.7%), indicating limited use of this option, presumably due to the substantial monitoring requirements (INR checks) and the growing availability of more convenient alternatives. Similarly, the use of ASA in three patients (9.7%) suggests a targeted approach for individuals with specific indications (for example, cardiovascular prevention), rather than as monotherapy for venous thrombotic events. It should also be noted that four patients (12.9%) did not receive any pharmacological anticoagulant treatment. This group may reflect complex clinical scenarios—such as patients with a high bleeding risk, limited prognosis, or explicit refusal of therapy—or situations in which the risk/benefit ratio of anticoagulation was deemed unfavorable. No patient was managed with UFH, indicating that current clinical practice generally favors a fixed-dose subcutaneous regimen (e.g., LMWH) over continuous UFH infusion, which is typically reserved for particularly unstable circumstances or cases requiring rapid dose adjustments. Overall, the findings demonstrate a preference for LMWH as the primary treatment strategy, with modest use of DOACs and other agents (FDP, ASA, VKA) based on each patient’s indications and clinical status. This variability in therapeutic choices likely reflects the complexity of clinical presentations and underscores the need for shared protocols or additional prospective data to determine the optimal strategy in specific patient subgroups. No statistically significant differences emerged between patients who died and those who survived, nor between those who developed PE and those who did not, indicating that IJVT severity may be multifactorial. Similarly, while active cancer is well-established as a risk amplifier for venous thromboembolism, sub-analyses did not detect clear laboratory differentiations, perhaps reflecting the small sample size or the heterogeneity of tumor types.

Overall, the data highlight the complex interplay of risk factors—malignancy, device use, and individual patient comorbidities—and the importance of tailored therapeutic strategies. They also reinforce the need for clinicians to maintain a high index of suspicion for IJVT, particularly in hospitalized patients with catheters and/or cancer, given its potential to progress to serious complications, including pulmonary embolism.

## 6. Limitations

Several limitations should be considered when interpreting these findings. First, the relatively small sample size may limit generalizability and the statistical power to detect significant differences in important clinical outcomes. Second, we did not systematically assess the influence of tumor stage, chemotherapy regimens, or other treatment factors that might affect thrombosis risk. Third, biomarker measurements were typically obtained at a single time point.

## 7. Conclusions

In this cohort, IJVT emerged as a clinically relevant entity among hospitalized patients, often occurring in individuals with active malignancy or in those who required a central venous access device. While device-related thrombosis was associated with distinct laboratory findings, no significant differences in mortality or pulmonary embolism rates were observed relative to non-CVAD cases. These results underscore the importance of vigilant monitoring, individualized therapeutic decision-making, and further prospective research to optimize strategies for preventing and managing IJVT, particularly in the complex context of cancer and catheter use. In our opinion, the present work should be considered a pilot study. Our results underline the need for larger, prospectively designed studies with adequate power to confirm and expand upon these findings. Particularly, future research should focus on developing device-specific diagnostic algorithms and prophylactic strategies.

## Figures and Tables

**Table 1:** Baseline and medical history characteristics of the enrolled patients.

Type of Data	Characteristic	N Patients	(%)
**Demographic Data**	Total Patients	31	100.0
	Gender (Male)	17	45.2
	Gender (Female)	14	54.8
**Comorbidities**	Device Use (Total)	11	35.5
	-CVC	4	12.9
	-Midline	4	12.9
	-PICC	3	9.7
	Congenital Thrombophilia	0	0
	Oral Contraceptive Use	1	3.2
	Diabetes	6	19.4
	Sepsis	6	19.4
	Hypertension	13	41.9
	Heart Failure	0	0
	Cerebrovascular Disease	3	9.7
	Active Malignancy	10	32.3
	Metastasis	5	16.1
	Previous DVT	2	6.5
	Previous Pulmonary Embolism	1	3.2
	Chronic Venous Disease	1	3.2
	Liver Disease	4	12.9
	Ischemic Heart Disease	3	9.7
	Atrial Fibrillation	5	16.1
	Psoriasis	1	3.2
	Previous bleeding	2	6.5
**Home Treatment**	VKA	2	6.5
	DOAC	2	6.5
	LMWH	3	9.7
	ASA	4	12.9

Abbreviations: aspirin, ASA; low molecular weight heparin, LMWH; direct oral anticoagulant, DOAC; vitamin K antagonist, VKA; deep venous thrombosis, DVT; central venous catheter, CVC; peripherally inserted central catheter, PICC.

**Table 2:** Laboratory tests of the enrolled patients. Values are expressed as mean ± standard deviation (SD).

Laboratory Finding	Mean ± SD
D-dimer (µg/mL)	3.2 ± 1.0
Platelets (×10^9^/L)	211.7 ± 75.4
AST (U/L)	25.0 ± 14.9
ALT (U/L)	22.0 ± 21.1
Creatinine (mg/dL)	1.6 ± 1.8
Total Bilirubin (mg/dL)	0.9 ± 1.0
CRP (mg/L)	8.2 ± 8.1

Abbreviations: aspartate aminotransferase, AST; alanine aminotransferase, ALT; C-Reactive Protein, CRP.

**Table 3:** Sub-analysis of basal characteristics between patients with vascular device on not.

	Device	No Device	t	*p*-Value
D-Dimer (µg/mL)	3.6 ± 0.8	2.1 ± 0.5	3.2	0.018 *
Platelets (×10^9^/L)	184.3 ± 53.6	249.0 ± 86.7	−2.1	0.044 *
AST (U/L)	24.4 ± 15.3	25.8 ± 15.0	−0.235	0.816
ALT (U/L)	14.8 ± 11.4	31.7 ± 27.4	−1.919	0.078
Creatinine (mg/dL)	1.4 ± 1.4	1.9 ± 2.3	−0.544	0.587
Total Bilirubin (mg/dL)	1.1 ± 1.3	0.5 ± 0.2	1.714	0.107
CRP (mg/L)	5.1 ± 5.6	12.5 ± 9.2	−2.335	0.033 *

Abbreviations: aspartate aminotransferase, AST; alanine aminotransferase, ALT; C-Reactive Protein, CRP. *statistically significant

**Table 4:** Sub-analysis of basal characteristics between alive and dead patients.

	Alive	Dead	t	*p*-Value
Platelets (×10^9^/L)	206.9 ± 72.8	248.3 ± 102.3	−0.678	0.560
AST (U/L)	24.2 ± 14.7	31.0 ± 18.5	−0.610	0.596
ALT (U/L)	20.1 ± 17.5	36.0 ± 43.3	−0.626	0.593
Creatinine (mg/dL)	1.7 ± 1.9	1.2 ± 0.5	0.862	0.409
Total Bilirubin (mg/dL)	0.9 ± 1.1	0.6 ± 0.1	1.098	0.284
CRP (mg/L)	7.5 ± 6.3	14.2 ± 17.6	−0.652	0.579

Abbreviations: aspartate aminotransferase, AST; alanine aminotransferase, ALT; C-Reactive Protein, CRP.

**Table 5:** Sub-analysis of basal characteristics between patients with pulmonary embolism or not.

	No PE	PE	t	*p*-Value
D-dimer (µg/mL)	2.9 ± 1.2	3.6 ± 0.7	−1.085	0.310
Platelets (×10^9^/L)	205.3 ± 79.4	187.2 ± 51.6	0.569	0.588
AST (U/L)	24.3 ± 16.2	20.5 ± 3.6	0.901	0.378
ALT (U/L)	17.8 ± 16.9	18.2 ± 10.6	−0.054	0.958
Creatinine (mg/dL)	1.6 ± 1.8	2.2 ± 2.8	−0.367	0.734
Total Bilirubin (mg/dL)	1.0 ± 1.2	0.4 ± 0.1	1.777	0.093
CRP (mg/L)	6.7 ± 6.2	8.7 ± 8.4	−0.451	0.677

Abbreviations: aspartate aminotransferase, AST; alanine aminotransferase, ALT; C-Reactive Protein, CRP.

**Table 6:** Sub-analysis of basal characteristics between patients with active cancer or not.

	No Active Cancer	Active Cancer	t	*p*-Value
Platelets (×10^9^/L)	200.0 ± 77.7	233.8 ± 69.5	−1.134	0.272
AST (U/L)	25.2 ± 16.5	24.5 ± 12.3	0.118	0.907
ALT (U/L)	21.5 ± 22.9	22.8 ± 18.5	−0.164	0.872
Creatinine (mg/dL)	2.0 ± 2.1	0.9 ± 0.4	2.04	0.056
Total Bilirubin (mg/dL)	0.9 ± 1.2	0.8 ± 0.7	0.240	0.812
CRP (mg/L)	8.9 ± 8.9	7.0 ± 6.5	0.596	0.557

Abbreviations: aspartate aminotransferase, AST; alanine aminotransferase, ALT; C-Reactive Protein, CRP.

**Table 7:** Types of treatment administered to patients after the detection of internal jugular vein thrombosis.

Type of Treatment	N (%)
VKA	3 (9.6%)
Dabigatran	0
Edoxaban	1 (3.2%)
Apixaban	3 (9.6%)
Rivaroxaban	0
LMWH	12 (38.7%)
FDP	5 (16.1%)
ASA	3 (9.6%)
UFH	0
No treatment	4 (12.9%)

Abbreviations: aspirin, ASA; low molecular weight heparin, LMWH; direct oral anticoagulant, DOAC; vitamin K antagonist, VKA; unfractioned heparin, UFH; fondaparinux, FDP.

**Table 8:** Clinical study outcomes.

Outcome	Number of Patients (N)	%
Bleeding	2	6.5%
Mortality	3	9.7%
Pulmonary embolism	5	16.1%

**Table 9:** Sub-analysis of basal characteristics between patients treated or not treated with antithrombotic therapy.

	Not Treated	Treated	t	*p*-Value
Platelets (×10^9^/L)	228.5 ± 97.8	208.6 ± 73.0	0.386	0.721
Hemoglobin (g/dL)	12.6 ± 0.8	12.0 ± 0.6	0.577	0.667
AST (U/L)	20.2 ± 12.5	25.8 ± 15.4	−0.793	0.465
ALT (U/L)	20.7 ± 25.5	22.2 ± 20.9	−0.109	0.919
Creatinine (mg/dL)	1.8 ± 1.6	1.6 ± 1.9	0.248	0.815
Total Bilirubin (mg/dL)	0.6 ± 0.3	0.9 ± 1.1	−1.004	0.329
CRP (mg/L)	13.1 ± 15.0	7.4 ± 6.3	0.747	0.506

Abbreviations: aspartate aminotransferase, AST; alanine aminotransferase, ALT; C-Reactive Protein, CRP.

## Data Availability

Data are available upon request from authors.
